# The Therapeutic Potential of Insulin Eye Drops in Neurotrophic Keratopathy: A Comprehensive Review

**DOI:** 10.3390/biomedicines13071657

**Published:** 2025-07-07

**Authors:** Roxana Scripcă, Sinziana Istrate, Emil Ungureanu, Ștefan Oprea, Nicoleta Anton, Madalina Boruga, Marius Alexandru Moga, Ancuța-Georgiana Onofrei

**Affiliations:** 1 Ophthalmology Department, Medicine Faculty, University Transilvania Brasov, Bulevardul Eroilor 29, 500036 Brașov, Romania; 2 BINE Ophthalmology Clinic, 020483 Bucharest, Romania; 3 Department of Anatomy, Carol Davila University of Medicine and Pharmacy, 050474 Bucharest, Romania; 4 Department of Ophthalmology, Gr. T. Popa University of Medicine and Pharmacy Iasi, 700115 Iasi, Romania; 5 Department of Toxicology, Victor Babeș University of Medicine and Pharmacy, 300041 Timișoara, Romania; 6 Department of Ophthalmology, Carol Davila University of Medicine and Pharmacy, 050474 Bucharest, Romania

**Keywords:** insulin eye drops, topical insulin, neurotrophic keratopathy, corneal ulcer

## Abstract

This review explores the potential role of topical insulin drops in corneal regeneration by analyzing the mechanism of action and clinical outcomes. Corneal integrity restoration is crucial for ocular surface healing. This review synthesizes the current literature on topical insulin for neurotrophic keratopathy (NK), highlighting its mechanism of action, therapeutic potential, and clinical outcomes. Recent studies report high rates of epithelial regeneration, suggesting that topical insulin may be an effective adjunct or alternative to conventional treatments. Further randomized controlled trials are needed to confirm its long-term efficacy and optimal dosing. **Methods:** Considering the limited regenerative capacity of the corneal epithelium in NK and the increasing interest in novel therapy, we review the existing literature to evaluate the role and extent of topical insulin’s contribution to corneal healing by applying the PICO framework, which allows for a clear and systematic approach to literature selection and evaluation. The literature search and study selection were conducted manually following PRISMA guidelines. **Conclusions:** Most of the studies resulting from the selection have small samples, and there is a lack of large, randomized clinical trials. The evidence reviewed in this study suggests that topical insulin is a promising therapy for promoting corneal healing in neurotrophic keratopathy. While clinical trials have demonstrated significant epithelial regeneration, optimal dosing and long-term safety require further investigation. Compared to conventional treatments such as autologous serum or growth factor therapy, insulin eye drops provide a cost-effective alternative. Additional research through controlled trials is needed to formulate standardized therapeutic protocols and verify long-term outcomes.

## 1. Introduction

The cornea exhibits dense sensory innervation, predominantly supplied by the ophthalmic branch of the trigeminal nerve, which contributes to epithelial turnover, neurotrophic support, reflex lacrimation, and the orchestration of wound healing mechanisms [1]. As seen in neurotrophic keratopathy (NK), damage to corneal nerves disrupts these mechanisms, leading to persistent epithelial defects and potential corneal perforation [2]. Emerging evidence suggests that topical insulin may facilitate corneal healing by stimulating epithelial proliferation, reducing inflammation, and restoring cellular homeostasis.

### 1.1. Anatomy of the Cornea

The anterior segment is composed of the conjunctiva, cornea, lens, ciliary body, iris, and aqueous humor. The cornea and sclera form the outer protective layer of the eye. The cornea is a transparent, avascular tissue that contributes approximately two-thirds of the eye’s refractive power (40–44 D) and has a 1.376 refractive index. The cornea is horizontally round, convex, and aspheric. The confines of the anterior curve are 7.8 mm, and the posterior curve is roughly 6.5 mm [3].

### 1.2. Corneal Histology

The corneal surface is composed of a multi-layered squamous epithelium that does not undergo keratinization, particularly at the periphery. Basal epithelial cells exhibit a polygonal morphology, transitioning into progressively flattened cells as they approach the superficial strata. Within the epithelial layers, immune-responsive Langerhans cells can be observed, typically identifiable by CD1a expression. Bowman’s layer contributes significantly to the anterior stroma. The central stroma, which constitutes approximately 90% of the corneal thickness, is acellular and contains uniformly spaced collagen fibrils interspersed with mucoproteins and glycoproteins—essential components responsible for maintaining corneal transparency. This layer measures approximately 8 to 14 microns in thickness and lacks regenerative capacity. Descemet’s membrane, synthesized by corneal endothelial cells, measures around 10–12 microns in adults and also does not regenerate after injury. The innermost layer, the endothelium, is composed of a single layer of flattened cells that play a vital role in maintaining corneal deturgescence through active ionic transport mechanisms, thereby ensuring tissue transparency [4].

### 1.3. Corneal Innervation and Sensation

The cornea receives extensive sensory innervation, accompanied by a smaller proportion of autonomic fibers. These nerve fibers originate primarily from the ophthalmic branch of the trigeminal nerve. At the corneal periphery, both unmyelinated C fibers and myelinated Aδ fibers penetrate the stroma. As they extend toward the central cornea, they lose their myelin sheath, a physiological adaptation that contributes to maintaining corneal transparency [2]. Corneal innervation plays a vital role in promoting tear secretion, blinking reflexes, and the release of trophic factors necessary for epithelial maintenance. When corneal nerves are damaged, these processes are disrupted, leading to reduced corneal sensitivity, persistent epithelial defects, and, in severe cases, corneal perforation [5]. Approximately 20% of the sensory fibers are mechanoreceptors that detect mechanical stimuli and transmit sharp pain signals through thin myelinated Aδ fibers. Around 70% are polymodal nociceptors that respond to chemical mediators (e.g., acetylcholine, prostaglandins, bradykinin), thermal, and mechanical stimuli via slow-conducting unmyelinated C fibers [6]. The remaining 10% are cold thermoreceptors that are activated by tear film evaporation or exposure to cold air or solutions and signal through both Aδ and C fibers [7].

### 1.4. The Involvement of Insulin in the Corneal Healing Process

Insulin is a hormone with an essential role in cellular metabolism, closely related to the insulin growth factor (IGF), which is capable of stimulating keratinocyte migration. At the ocular surface, insulin stimulates the proliferation and migration of corneal epithelial cells and the synthesis of growth factors involved in tissue regeneration and significantly reduces inflammatory biomarkers [8]. The mechanism by which insulin contributes to the regeneration of the corneal epithelium is not fully understood, and the optimal dose has not yet been established in studies. Bartlett et al. applied eye drops of insulin in concentrations of 100 IU/mL to eight healthy eyes and demonstrated the safety of topical insulin in the form of drops [9].

The Mackie classification (Table 1) stages the severity of the lesions as follows [10].

## 2. Materials and Methods

For this review, a systematic search was performed in PubMed, Embase, Scopus, and Web of Science Core Collection using the terms “Insulin Eye Drops” OR “Topical Insulin” AND “Neurotrophic Keratopathy” OR “Corneal Ulcer”. A time restriction was applied to include only studies published between 1 January 2020 and 1 January 2025. This period was chosen to capture the most recent clinical evidence and methodological advances in the use of topical insulin for neurotrophic keratopathy. The final literature search was conducted on 31 March 2025, as predefined in the registered review protocol (PROSPERO ID: CRD420251043823).

Only peer-reviewed, full-text articles published in English and involving human subjects were considered. Filters were applied to exclude animal studies, in vitro experiments, editorials, and non-peer-reviewed publications.

- PubMed: The search yielded 108 records. After removing 18 duplicates, 90 titles and abstracts were screened. Of these, 35 full-text articles were assessed, and 12 studies were included in the final qualitative synthesis based on predefined eligibility criteria.
- Embase (via Ovid): The Ovid MEDLINE search initially yielded 61 results. After applying filters and reviewing the exportable records, 30 valid articles were retrieved and included for screening based on title and abstract. The discrepancy between the initial search count and the number of records analyzed was due to platform limitations and the removal of duplicate or incomplete entries. Fifteen studies were considered.
- Scopus: The initial search identified 63 articles. After manual screening of titles and abstracts, 23 potentially relevant articles were selected. Cross-referencing with the PubMed dataset led to the exclusion of 12 duplicates, leaving 11 unique studies for consideration.
- Web of Science Core Collection: A total of 17 studies were initially retrieved. After applying the eligibility criteria, specifically targeting studies addressing topical insulin in NK, 6 studies were excluded due to a lack of direct reference to NK, focus on unrelated pathologies, or use of animal models. Ultimately, 11 studies were deemed eligible for inclusion. One case report (study no. 8) was retained despite not explicitly diagnosing NK, as the clinical context of long-standing, refractory corneal ulcer suggested a neurotrophic etiology. This inclusion aligned with the review protocol, which allowed for clinically inferred relevance even when terminology varied.

All databases were searched independently. The results were deduplicated and screened according to standardized inclusion and exclusion criteria. The overall search and selection process adhered to PRISMA guidelines (see also Table 2, PRISMA flow summary).

### 2.1. Inclusion Criteria

- Clinical studies (RCTs, observational, case series) evaluating the effect of topical insulin on neurotrophic keratopathy.
- Preclinical studies (in vitro, animals) exploring the mechanism of action of insulin in corneal healing.
- Meta-analyses and systematic reviews synthesizing relevant data on ophthalmic insulin.
- Clinical guidelines mentioning insulin as a possible therapeutic option.

### 2.2. Exclusion Criteria

**Figure 1:**
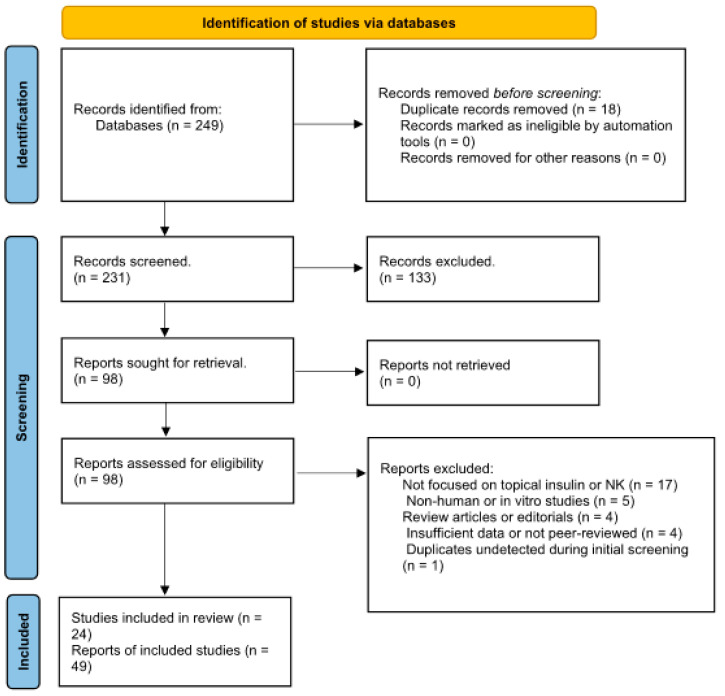
PRISMA flow diagram for (“Insulin Eye Drops” OR “Topical Insulin”) AND (“Neurotrophic Keratopathy” OR “Corneal Ulcer”).

- Studies analyzing systemic insulin without ophthalmological applicability.
- Studies on other corneal conditions not relevant to neurotrophic keratopathy.
- Letters to the editor, opinions, and articles without peer review.
- The literature search and study selection followed PRISMA guidelines (see Figure 1), and no automated screening tools (e.g., Rayyan, Covidence) were used.

### 2.3. Study Selection Process

All search results were reviewed manually by the first author, with titles and abstracts screened for relevance. Full texts were then assessed against eligibility criteria. Duplicates were removed. Discrepancies were resolved by consensus. No automation tools (e.g., Rayyan or Covidence) were used.

### 2.4. Data Extraction

Key data were extracted from the included studies into structured tables. Extracted variables included the following: study design, number of patients, insulin concentration and administration frequency, treatment duration, primary outcomes (e.g., healing time, adverse effects), and conclusions.

### 2.5. Risk of Bias Assessment

Due to the heterogeneity and small size of most included studies, no formal risk-of-bias tool (e.g., ROBINS-I or Cochrane Risk of Bias Tool) was applied. However, study limitations, including sample size and lack of control groups, were critically discussed in Section 4.

The risk of bias in the included non-randomized studies was assessed using the ROBINS-I tool. This evaluation systematically addressed the seven key domains outlined by the ROBINS-I framework: confounding, selection of participants, classification of interventions, deviations from intended interventions, missing data, measurement of outcomes, and selection of reported results. Most studies demonstrated a moderate risk of bias, particularly due to small sample sizes, lack of control groups, and potential confounding variables not being adequately accounted for. The classification of interventions and outcome measurement domains generally presented low risk, indicating clear definitions of interventions and consistent reporting of clinical endpoints. However, concerns remained regarding selection bias and the potential for selective outcome reporting in studies with limited transparency or short follow-up periods. No automation tools were used in the risk of bias assessment process, and evaluations were conducted manually.

## 3. Results

### 3.1. Study Selection

Following comprehensive searches across PubMed, Embase (via Ovid), Scopus, and Web of Science, a total of 92 records were initially identified as potentially eligible, based on predefined inclusion criteria. After title and abstract screening and the removal of duplicates, 49 studies were retained and reported in the PRISMA flow diagram as eligible for full-text evaluation. However, a detailed full-text review and data extraction process revealed further duplications, overlapping patient cohorts, and instances of redundant reporting across multiple publications. Consequently, 24 unique and methodologically eligible studies were ultimately included in the final qualitative synthesis (Table 3).

### 3.2. Comparison (C): Standard Treatments for Neurotrophic Keratopathy (NK)

A comparative analysis of included studies highlights the promising efficacy of topical insulin in the treatment of neurotrophic keratopathy (NK). Most of the clinical studies and case reports reviewed report high rates of re-epithelialization and improvement of corneal transparency without significant adverse effects [14]. Reported studies indicate a healing rate between 75% and 90% in patients treated with topical insulin (Table 4). No major adverse effects have been reported following the use of topical insulin [21]. The treatment duration ranged from 7 days to 8 weeks, depending on the severity of corneal lesions and response to therapy [15]. Studies suggest that the combination treatment (insulin + Hyper-CL therapeutic lenses) improves absorption and clinical effects. One possible explanation for the improved outcomes observed with insulin and therapeutic lenses is enhanced bioavailability and prolonged contact time with the corneal epithelium, leading to increased cellular uptake and sustained release of insulin over time [20]. Neurotrophic keratopathy (NK) is a challenging condition characterized by impaired corneal healing due to sensory nerve damage. Conventional treatments aim to restore corneal integrity, reduce inflammation, and promote epithelial regeneration [2,15].

Large-scale randomized clinical trials are needed to establish the optimal dose and exact duration of treatment. Future research should explore possible synergies with other therapies to maximize clinical benefits. In conclusion, topical insulin emerges as a promising and affordable treatment for NK, with a high safety profile and favorable outcomes in corneal epithelial regeneration.

### 3.3. Comparison of Topical Insulin vs. Standard Treatments for Neurotrophic Keratopathy

Topical insulin has emerged as a novel therapeutic option for neurotrophic keratopathy (NK), particularly in patients who have shown suboptimal response to conventional therapies such as artificial tears, punctal occlusion, therapeutic contact lenses, or autologous serum (Table 5). While standard treatments aim to restore surface hydration and reduce epithelial trauma, they often fail to stimulate regeneration of the corneal epithelium. In contrast, topical insulin appears to enhance epithelial proliferation and wound healing by activating insulin and IGF-1 receptors, promoting epithelial migration and mitogenesis.

The reviewed studies suggest that topical insulin may offer comparable or even superior outcomes in terms of epithelial defect resolution and time to healing, especially in moderate to severe NK (Mackie stages 2–3). However, direct comparative trials are scarce, and most included studies lacked control groups. Despite these limitations, the preliminary evidence indicates that topical insulin could complement or, in select cases, replace conventional treatments, especially when regenerative stimulation is the primary therapeutic goal.

### 3.4. Characteristics of Included Studies

The included studies collectively reported on a total of 94 patients treated with topical insulin for neurotrophic keratopathy. Insulin concentrations ranged from 1 IU/mL, being the most frequently used dosage, typically administered three to four times daily. Treatment durations varied from 7 days to 12 weeks, depending on the severity of the epithelial defect and the patient’s clinical response. It is important to emphasize that the 100 IU/mL concentration was used exclusively to evaluate ocular tolerability and potential adverse effects rather than therapeutic efficacy.

### 3.5. Efficacy Outcomes

Most clinical studies reported significant rates of re-epithelialization, ranging from 75% to 90% after 6–8 weeks of treatment. Improvements in corneal transparency, ocular surface integrity, and patient-reported symptoms were consistently noted. One study [21] found that combining insulin with Hyper-CL therapeutic contact lenses accelerated epithelial recovery compared to insulin alone.

### 3.6. Safety and Tolerability

No major adverse events were reported across the included studies. Mild transient discomfort upon installation was noted in two patients but did not lead to discontinuation of therapy. None of the reviewed studies documented long-term adverse effects such as corneal neovascularization, fibrosis, or decreased transparency, although most follow-up periods were limited to ≤8 weeks.

The therapeutic outcomes reported across the included studies were predominantly descriptive, reflecting the observational or exploratory nature of the available literature. Most studies involved small sample sizes and lacked control groups, limiting the use of formal effect estimates or statistical comparisons. Nevertheless, consistent improvements in corneal epithelial healing were documented in response to topical insulin, either as monotherapy or in combination with therapeutic contact lenses. The table below summarizes key findings from each study, including intervention characteristics, treatment duration, and re-epithelialization rates. While no confidence intervals or comparative statistics were available, the reported efficacy and absence of major adverse effects suggest a promising clinical utility of topical insulin in neurotrophic keratopathy.

### 3.7. Limitations in Evidence

While preliminary results are encouraging, the small sample sizes, lack of control groups, and absence of randomized controlled trials (RCTs) limit the generalizability of the findings. Additionally, the short duration of follow-up in all studies prevents firm conclusions regarding the long-term safety and efficacy of topical insulin therapy. No sensitivity analysis was performed due to the descriptive nature and limited number of included studies.

## 4. Discussion

The largest clinical trials of ophthalmic insulin have involved 37 patients, highlighting the need for research on larger sample sizes [14,15]. Case studies examine the effects of insulin on 1–4 patients [13]. The standard dose used in most clinical trials is 1 unit/mL, administered 3 to 4 times daily [15]. The duration of treatment varies between 6 and 12 weeks, with most studies reporting epithelial healing after 6–8 weeks [21]. Patients treated with insulin and therapeutic lenses had faster results (6 weeks vs. 8–10 weeks in insulin therapy alone) [15]. There are no long-term clinical studies yet (more than 3 months of follow-up) [14]. Although most studies included in this review report high rates of re-epithelialization and the absence of major adverse effects associated with the use of topical insulin, the duration of post-treatment follow-up is limited. The longest follow-up period identified was 2 months (8 weeks) in the study by Khilji et al. (2023), in which a patient with post-herpetic neurotrophic keratopathy (NK) was treated and followed for 2 months [13].

In other studies, follow-up durations ranged from 7–45 days in the study of Soares et al. (2022) [14], 6 weeks in Eleiwa et al. (2024) [15] and Eleiwa et al. (2025) [18], and 8 weeks in the studies by Mancini et al. (2024) [20] and Giannaccare et al. (2024) [21].

The lack of long-term studies (more than 3–6 months) limits the ability to assess late effects of topical insulin, such as possible corneal structural changes over time, risk of angiogenesis or corneal fibrosis, and potential decrease in efficacy with prolonged use [14,15,20].

Current data suggest that topical insulin is safe and well tolerated, but studies with extended follow-up (>6 months–1 year) are needed to confirm the safety profile and possible late adverse effects [20,21]. Future studies should include detailed clinical follow-up and histological analyses to determine long-term effects on the cornea [14,17]. The evidence reviewed in this study suggests that topical insulin is a promising therapy for promoting corneal healing in neurotrophic keratopathy [14,15,21]. While clinical studies have demonstrated significant epithelial regeneration, optimal dosing and long-term safety require further investigation [14,17]. Compared to conventional treatments such as autologous serum or growth factor therapy, insulin eye drops provide a cost-effective alternative with the potential for widespread application [11,21]. Large-scale, randomized controlled trials are necessary to establish optimal dosing, assess long-term safety, and determine the role of topical insulin as a standardized therapy for neurotrophic keratopathy [43].

## 5. Study Limitations and Future Research Prospects

Although the current results suggest that topical insulin may represent a promising therapeutic strategy for neurotrophic keratopathy (NK), there are still insufficiently explored aspects, especially regarding the safety of long-term use and potential adverse effects [14,17]. One of the main concerns is related to the possibility of corneal angiogenesis and stromal fibrosis, phenomena that could compromise corneal transparency and, implicitly, visual function [11,43].

### 5.1. Possibility of Corneal Angiogenesis

The cornea is naturally an avascular tissue, and maintaining this status is essential for visual function. Insulin is known as an anabolic factor with an essential role in cellular metabolism and, in certain pathological contexts, has been associated with the regulation of the expression of vascular endothelial growth factor (VEGF), a cytokine essential in neovascularization processes [44]. In diabetic patients, hyperinsulinemia has been correlated with increased levels of VEGF, thus contributing to proliferative diabetic retinopathy [45]. This observation raises the question of whether prolonged use of insulin at the corneal level could indirectly stimulate angiogenesis, thus affecting corneal transparency. However, in the clinical studies analyzed, no such effects were reported. It should be noted, however, that the maximum duration of patient monitoring was approximately 8 weeks, which does not allow sufficient assessment of the risk of neovascularization in the long term. Therefore, prospective studies with extensive monitoring are needed to determine whether topical insulin can have an angiogenic effect in the cornea or whether it remains safe for chronic use [11,17].

### 5.2. Possibility of Corneal Fibrosis

Another aspect that requires further investigation is the potential risk of stromal fibrosis. Insulin plays a fundamental role in cellular proliferation and migration and interacts with key modulators such as transforming growth factor-beta (TGF-β) and platelet-derived growth factor (PDGF), both of which are deeply involved in extracellular matrix remodeling and fibrogenesis [8,46]. If topical insulin excessively stimulates the activity of corneal fibroblasts, it could result in irregular collagen deposition, compromising corneal transparency and reducing visual acuity. Although no direct clinical evidence currently supports this hypothesis, the absence of detailed histological assessments in current studies restricts our understanding of insulin’s effects on the stromal layer. Therefore, future investigations should include advanced histological and imaging techniques such as in vivo confocal microscopy and anterior segment OCT to monitor stromal remodeling after prolonged insulin use [17].

### 5.3. Limitations of the Current Studies

Although current data support the efficacy of topical insulin in promoting corneal epithelial regeneration, it is important to acknowledge several methodological limitations of the reviewed studies. The maximum post-treatment follow-up reported was 8 weeks, as seen in studies such as those by Khilji et al. (2023) [13] and Mancini et al. (2024) [20], which is insufficient to evaluate potential long-term outcomes. Most clinical studies included relatively small cohorts, typically ranging from 8 to 21 patients, which reduces the statistical power and generalizability of findings [14,21]. Furthermore, much of the current evidence is derived from observational and retrospective analyses, with a notable lack of randomized controlled trials to validate the efficacy and safety of insulin therapy for neurotrophic keratopathy [17,19]. In addition, considerable heterogeneity exists in insulin dosing regimens (ranging from 1 IU/mL to 100 IU/mL), frequency of administration, and treatment duration, making it challenging to establish a standardized therapeutic protocol [15,20].

### 5.4. Directions for Future Research

The review of the therapeutic potential of insulin eye drops in neurotrophic keratopathy adds significant value to the existing literature by providing an in-depth overview of the current state of insulin eye drop research. While several narrative and systematic reviews have addressed the role of growth factors and autologous serum in the management of neurotrophic keratopathy (NK), few have focused exclusively on topical insulin as a therapeutic agent. This review adds value to the existing literature by providing a focused and updated synthesis of clinical and preclinical evidence published between 2020 and 2025, examining all mechanisms of action as well as therapeutic outcomes associated with topical insulin in NK. The review critically discusses methodological limitations of current studies, such as small sample sizes, lack of control groups, and the absence of randomized controlled trials (RCTs). This critical perspective is essential for guiding future research directions and creating study designs in this area. Highlighting the short duration of follow-up in existing studies, the review raises important questions about the long-term safety and efficacy of topical insulin therapy. This focus on long-term outcomes is often overlooked in other reviews, making this work particularly valuable to clinicians and researchers. The review examines variations in dose, frequency of administration, and treatment duration across studies. Addressing these inconsistencies paves the way for the development of standardized treatment protocols, which are crucial for clinical practice and future research. The review not only summarizes existing knowledge but also identifies gaps in the literature, encouraging further investigation. This is vital for advancing the field and ensuring that future studies build on the findings and limitations highlighted in this review. In summary, this review contributes to the existing body of literature by providing a comprehensive, critical, and forward-looking review of the therapeutic potential of insulin eye drops in neurotrophic keratopathy, thereby increasing understanding and guiding future research efforts. Thus, the present review contributes a targeted and critical perspective to the evolving field of ocular surface regeneration, with the aim of supporting the development of standardized therapeutic protocols involving topical insulin.

To strengthen the position of topical insulin as a safe and effective therapy for NK, future studies should include randomized clinical trials (RCTs) with larger patient samples to validate the efficacy and safety of the therapy [17]. Extended follow-up (>6 months–1 year) is also necessary to detect possible long-term adverse effects, including the risk of neovascularization or fibrosis [15]. Histological and imaging studies are recommended to evaluate the effects of insulin on corneal stromal architecture [20]. Additionally, comparison with standard therapies—such as nerve growth factor (NGF), autologous serum, or corneal neurotization—should be pursued to identify the specific advantages and limitations of each therapeutic approach [21].

## 6. Conclusions

In conclusion, topical insulin represents an innovative and affordable therapeutic option for neurotrophic keratopathy, offering a cure rate between 75% and 90%, according to available studies [14]. However, the safety of long-term use remains uncertain, and potential effects on angiogenesis and fibrogenesis need to be explored further [44]. Until these findings are confirmed through randomized controlled trials, topical insulin should be applied in clinical practice with caution and close patient monitoring to ensure maximum therapeutic benefit and minimize risk [20].

## Figures and Tables

**Table 1:** The Mackie classification [10].

Stage	Clinical Features
Stage 1	dry and cloudy corneal epithelium, the presence of superficial punctate keratopathy and edema
Stage 2	recurrent and/or persistent epithelial defects with an oval or circular shape in the upper half of the cornea
Stage 3	corneal ulcer with stromal involvement, stromal melting phenomena, and progression to corneal perforation

**Table 2:** PRISMA flow summary.

Search Step	Number of Studies
Records identified through PubMed	108
Records after duplicates removed (PubMed)	90
Full-text articles assessed (PubMed)	35
Studies included (PubMed)	12
Records identified through Embase	61
Full-text articles assessed (Embase)	30
Studies included (Embase)	15
Records identified through Scopus	63
Potentially relevant articles (Scopus)	23
Duplicates excluded (Scopus)	12
Unique studies included (Scopus)	11
Records identified through Web of Science	17
Excluded after screening (Web of Science)	6
Studies included (Web of Science)	11

**Table 3:** Topical insulin studies—Structured table from PubMed, Embase, Word of Science, and Web of Science with 32 titles analyzed: one animal model study; 31 human studies, of which 14 are reviews, 6 are case reports, 10 are clinical studies, and one is a meta-analysis.

Nr.	Title	Study Type	Number of Eyes	Insulin Dosage	Treatment Period	Results	Conclusions
1	The role of topical insulin in ocular surface restoration: A review [11].	Review	N/A	N/A	N/A	N/A	Insulin has significant potential in ocular surface regeneration. The main mechanisms include stimulation of cell proliferation, epithelial migration, and anti-inflammatory effects. Further clinical studies are needed to determine optimal dosing and standardized protocols.
2	Topical Insulin in Neurotrophic Keratopathy: A Review of Current Understanding of the Mechanism of Action and Therapeutic Approach [12].	Review	N/A	N/A	N/A	N/A	Explains in detail the mode of action of insulin on the ocular surface. Insulin enhances epithelial cell migration and increases the expression of neurotrophic factors. Discusses the use of insulin in combination with other therapies, such as autologous serum and growth factors.
3	Neurotrophic Keratopathy and Topical Insulin Therapy: A Case Report [13].	Case study	1 eye	1 IU/mL of regular insulin in 10 mL of artificial tears with polyvinyl alcohol	8 weeks		Reports a successful clinical case in which topical insulin was used to treat severe neurotrophic corneal lesions. Epithelial healing was accelerated, and the patient had significant improvement in symptoms.
4	Topical Insulin—Utility and Results in Refractory Neurotrophic Keratopathy in Stages 2 and 3 [14].	Clinical study	21 eyes	1 IU/mL, applied 4 times/day	7 to 45 days to complete reepithelialization	Complete epithelial healing in most patients after 6–8 weeks.	Ophthalmic insulin was used for patients with refractory neurotrophic keratopathy (stages 2 and 3). Positive results: accelerated epithelial healing and improved quality of life of patients. No major adverse effects were reported.
5	Topical insulin in neurotrophic keratopathy after diabetic vitrectomy [15].	Clinical study	37 eyes	Concentration of 50 IU/mL of insulin. One unit of insulin per drop from the 50 IU/mL solution in artificial tears	6 weeks	Significant improvement in corneal re-epithelialization after 4 weeks, with results maintained at 6 weeks.	It reviews the use of ophthalmic insulin in patients with neurotrophic keratopathy secondary to vitrectomy for diabetic retinopathy. The results were positive, but with variation between patients. It recommends further studies for validation.
6	Physicochemical and microbiological stability of insulin eye drops in an artificial tear vehicle used in the treatment of refractory neurotrophic keratopathy [16].	Experimental study (pharmaceutical stability)	N/A	N/A	N/A	N/A	Analyzes the stability of topical insulin in artificial tears and ophthalmic solutions. Insulin remains chemically and microbiologically stable in an artificial environment. Recommends specific formulations to optimize bioavailability.
7	Topical Insulin for Neurotrophic-Related Epithelial Defects: Where Do We Stand? A Systematic Review [17].	Review	N/A	N/A	N/A	N/A	It is the most comprehensive systematic review of topical insulin in the treatment of neurotrophic epithelial defects. It compares the effectiveness of insulin with other treatments such as autologous serum and growth factors. Overall conclusion: insulin has great potential, but studies are heterogeneous and require standardization.
8	Topical insulin in pediatric neurotrophic keratopathy associated with CIPA syndrome [18].	Pediatric clinical study	2 eyes	1 IU/mL, applied 3–4 times/day	6–8 weeks, 2 years follow up	Significant epithelial healing after 6 weeks, with progress maintained at 8 weeks.	Reports positive results in the treatment of neurotrophic keratopathy in children with CIPA (congenital insensitivity to pain with anhidrosis). Ophthalmic insulin accelerated the healing of epithelial defects in children. No significant adverse effects were reported.
9	The Utilization of Topical Insulin for Ocular Surface Diseases: A Narrative Review [19].	Review	N/A	N/A	N/A	N/A	Discusses the use of topical insulin not only in KN but also in other ocular surface diseases (e.g., keratitis). This is not a systematic review but provides a broad context for the use of ophthalmic insulin. Concludes that randomized trials are needed for validation.
10	Topical insulin used alone or in combination with drug-depository contact lens for refractory cases of neurotrophic keratopathy [20].	Clinical study	9 eyes	1 IU/mL, applied 4 times/day	12 weeks	Patients treated with insulin alone showed epithelial healing after 8–10 weeks, and those with insulin + therapeutic lenses had improvements after 6 weeks.	Analyzes the use of insulin with therapeutic lenses for the treatment of refractory KN. Conclusion: combined treatment improves healing time and epithelial stability.
11	Combined Use of Therapeutic Hyper-CL Soft Contact Lens and Insulin Eye Drops for the Treatment of Recalcitrant Neurotrophic Keratopathy [21]	Case report	1 eye	Humalog 1 IU/mL with artificial tears based on a hydroxypropyl guar.	20 days	Complete closure after 20 days of therapy	Confirms the benefits of combining insulin with therapeutic lenses. The treatment was effective in cases of KN resistant to other therapies.
12.	Insulin eye drops for treating corneal ulcers in a non-diabetic patient: regarding a case [22]	Case report	1 eye	Insulin formulation 1 IU/mL four times a day	3 months	2 months clear regression of the lesion size	Insulin drop formulation is effective and has an absence of toxicity in the treatment of a non-diabetic patient with a post-caustic corneal ulcer refractory to conventional therapy.
13.	Neurotrophic keratopathy: Update in diagnosis and management [23]	Review	N/A	N/A	N/A	N/A	There are several ongoing studies evaluating novel, promising strategies that can expand the therapeutic options for NK (topical insulin included).
14.	Recent United States Developments in the Pharmacological Treatment of Dry Eye Disease [24]	Review	N/A	N/A	N/A	N/A	DED, an umbrella term encompassing multiple etiologies, is a field that is experiencing the emergence of innovative solutions (insulin drops).
15.	Comparative study between topical insulin, autologous serum, and honey-based eye drops as adjunctive treatment in resistant corneal ulcer [25]	Comparative study	15 eyes	Topical formulation from Actrapid; 100 IU/mL with saline serum, dosage 1 IU/mL, four times a day	4 weeks	2–4 weeks	This study demonstrated the role of topical insulin, autologous serum, and honey-based eye drops when used as adjunctive methods added to culture-based medical therapy in the management of resistant corneal ulcers and showed that they were comparable to each other.
16.	Neurotrophic keratopathy: General features and new therapies [26].	Review	N/A	N/A	N/A	N/A	Topical insulin is well-tolerated, does not cause ocular side effects, and is accessible for use in cases like NK. To achieve substantial advances in prevention, early detection, and effective treatment of NK, further research targeting the loss of corneal sensation is needed to better understand the condition.
17.	Limitations and advances in new treatments and future perspectives of corneal blindness [27]	Review	N/A	N/A	N/A	N/A	Novel therapeutic strategies using growth factors, anabolic agents, new promitotic and anti-inflammatory drugs, combined with delivery systems, or corneal or LG genetic reprogramming of cells in association or not with corneal tissue reengineering can reduce the need for corneal transplantation and may function as adjuvants, providing customized therapies supporting more stable and long-lasting therapies for corneal transparency.
18.	Insulin eye drops for neurotrophic keratitis [28]	Case report	2 eyes	Insulin eye drops (0.1 mL of 100 IU/mL of insulin aspart, fast-acting insulin in 10 mL of PEG 400-Propylene Glycol eye drops) 4 times/day	25 days	8–12 days	Topical insulin drops may be used to manage NK unresponsive to conventional treatments.
19.	Topical insulin for refractory persistent corneal epithelial defects [29]	prospective non-randomized hospital-based study	21 eyes	1 IU/mL	23 days median	7–114 days	Topical insulin can promote and accelerate corneal reepithelization of refractory PEDs. It also offers many other advantages, including excellent tolerance, availability, and cost-effectiveness.
20.	Insulin eye drops improve corneal wound healing in STZ-induced diabetic mice by regulating corneal inflammation and neuropeptide release [30]	Animal model study	N/A	N/A	N/A	N/A	The results indicated that insulin eye drops may accelerate corneal wound healing and decrease inflammatory responses in diabetic mice by promoting nerve regeneration and increasing levels of neuropeptides SP and CGRP.
21.	Efficacy of Topical Insulin Therapy for Chronic Trophic Ulcers in Patients with Leprosy: A Randomized Interventional Pilot Study [31]	Randomized Controlled Trial					Topical insulin therapy may be a safe, efficacious, cheap, and easily available treatment option in CTUs among patients with leprosy.
22.	Current and Emerging Therapeutic Strategies for the Management of Neurotrophic Keratitis [32]	Review	N/A	N/A	N/A	N/A	Treatments that aim to promote cornea epithelial and nerve regeneration and that limit further damage from infection and friction on a friable corneal surface are promising.
23.	The management of neurotrophic keratitis [33]	Review	N/A	N/A	N/A	N/A	Many novel treatments based on agents that stimulate nerve regrowth are now available to treat NK. Improvement in neurotization procedures could also address advanced stages of this disease with surgery.
24.	Randomized controlled trial on effects of topical insulin compared to artificial tears and normal saline on tear inflammatory mediator levels and clinical parameters in diabetics with dry eye disease [34]	Randomized Controlled Trial	30 patients	topical insulin 25 IU/mL in saline solution	4 weeks	N/A	In this study, treatment with topical insulin resulted in the greatest reduction in all ocular biomarkers of inflammation tested, as compared to artificial tears and normal saline. Topical insulin was the most effective in improving clinical parameters of dry eye disease in diabetics, although all showed significant improvements, suggesting that regular use of topical insulin may be beneficial in treating diabetic dry eye disease.
25.	Advances in the Medical Management of Neurotrophic Keratitis [35]	review	N/A	N/A	N/A	N/A	Recent advances in the development of therapeutics for NK have provided substances such as recombinant human nerve growth factor (Cenegermin), currently approved for clinical use in the United States and Europe, as well as other promising therapeutic options that are in pre-clinical development, such as thymosine β4, connexin43 inhibitors, and artificial extracellular matrix components.
26.	The molecular basis of neurotrophic keratopathy: Diagnostic and therapeutic implications. A review [36]	Review	N/A	N/A	N/A	N/A	Adrenergic c-AMP- and cholinergic c-GMP-dependent responses are initiated after corneal insult, triggering epithelial activation, mitosis, and cell proliferation. NFs, especially NGF, BDNF, and GNDF, as well as neuromediators, play crucial roles in ocular surface integrity, but more importantly, they are involved in epithelial wound healing.
27.	Neurotrophic keratopathy: An updated understanding [37]	NK Study Group	N/A	N/A	N/A	N/A	Updated definition and staging system that provides clinicians with the necessary information to diagnose and treat NK at an early stage before it becomes a sight-threatening disorder.
28.	The Performance of Topical Insulin in Persistent Corneal Epithelial Defects and Persistent Corneal Ulcers—A Case Series [38]	Case reports	29 patients	25 IU/mL three times per day	42 days	42 days	Topical insulin may be considered as a treatment option in complicated cases refractory to conventional treatment, but outcomes may be less favorable than previously reported.
29.	Efficacy of treatments for neurotrophic keratopathy: a systematic review and meta-analysis [39]	Meta analysis	N/A	N/A	N/A	N/A	All specific treatments had a better percentage of complete healing than non-specific treatments, i.e., mainly lubricants, improving the percentage of complete healing with similar results.
30.	Neurotrophic Keratitis Following Vitrectomy Surgery: A Case Report [40]	Case report	1 eye	topical insulin (1 IU/mL every six hours).	2 weeks	2 weeks	Prompt diagnosis and tailored management, including the use of advanced therapies like autologous serum, can mitigate progression and improve outcomes.
31.	Neurotrophic keratitis: inflammatory pathogenesis and novel therapies [41]	Review	N/A	N/A	N/A	N/A	The lack of nerve-derived neuromediators and corneal-released neuropeptides, neurotrophins and neurotrophic factors in neurotrophic keratitis leads to a decrease in trophic supply to corneal cells in addition to a decrease in afferent signaling to the brain. Lately, nerve growth factor in special gained emphasis as a treatment strategy targeting the disease mechanism.
32.	New Pharmacological Approaches for the Treatment of Neurotrophic Keratitis [42]	review	N/A	N/A	N/A	N/A	Supported by evidence in the review, topical insulin is becoming more widely used in NK and is manufactured locally rather than being produced commercially.

**Table 4:** Comparative analysis of clinical Studies on topical insulin in neurotrophic keratopathy.

Study	Number of Patients	Insulin Dose	Period of Treatment	Cure Rate	Comments
Topical Insulin-Utility [14]	10	1 IU/mL, 4×/day	8 weeks	Complete epithelial healing in 80% of patients after 6–8 weeks	Effective for refractory KN
Insulin in KN post-vitrectomy [15]	18	1 IU/mL, 3×/day	6 weeks	Significant improvement in 85% of patients after 6 weeks	Positive response in vitrectomy patients
Insulin in pediatric KN [18]	4	1 IU/mL, 3–4×/day	6 weeks–8 weeks	Epithelial healing in 75% of patients after 6–8 weeks	Promising results in children
Topical insulin used alone or in combination with drug-depository contact lens for refractory cases of neurotrophic keratopathy [20]	12	1 IU/mL, 4×/day	12 weeks	Epithelial healing in 90% of patients after 6–10 weeks	Combination therapy accelerates healing
Combined Use of Therapeutic Hyper-CL Soft Contact Lens and Insulin Eye Drops for the Treatment of Recalcitrant Neurotrophic Keratopathy [21].	8	1 IU/mL, 4×/day	8 weeks	Epithelial healing in 75% of patients after 6–8 weeks	Hyper-CL lenses improve epithelial stability
Neurotrophic Keratitis Following Vitrectomy Surgery: A Case Report [40]	1	1 IU/mL every six hours	2 weeks	Corneal ulcer healing	Treatment: fortified amikacin (40 mg/mL), fortified vancomycin (5%), and topical insulin (1 IU/mL every six hours)
Neurotrophic Keratopathy and Topical Insulin Therapy: A Case Report [13]	1	insulin drops six times a day (prepared by mixing 1 IU/mL	4 weeks	NK secondary to prior herpetic keratitis	A case of NK secondary to prior herpetic keratitis. Treatment: oral acyclovir 400 mg twice a day, topical acyclovir ointment 5% five times a day, and topical insulin drops six times a day (prepared by mixing 1 IU/mL of regular insulin in 10 mL of artificial tears with polyvinyl alcohol) for a month.
Topical Insulin-Utility and Results in Refractory Neurotrophic Keratopathy in Stages 2 and 3 [14].	21 eyes	1 unit per mL	Four times per day and was continued until the persistent epithelial defect (PED) or ulcer resolved.	Refractory neurotrophic keratopathy (NK) in stages 2 and 3 treated with topical insulin	Retrospective analysis of eyes with NK in stages 2 and 3 refractory to standard medical and/or surgical treatment
Insulin eye drops for treating corneal ulcer in a non-diabetic patient: regarding a case [22]	1	50 IU/mL (1 IU/drop) with a dosage schedule of 1–2 drops/4 times daily.	Two months	Two months later, a clear regression of the lesion size was found, and no problem of toxicity has been detected; in treatment for three months; and has completely recovered corneal epithelium	Insulin drop formulation with effectiveness and absence of toxicity in a non-diabetic patient with a post-caustic corneal ulcer
The Performance of Topical Insulin in Persistent Corneal Epithelial Defects and Persistent Corneal Ulcers—A Case Series [38]	29 patients	25 IU/mL × 3 times per day Insulin drops were prepared by diluting fast-acting insulin with 0.9% normal saline, resulting in a concentration of 25 IU per milliliter or 0.5 IU per drop	42 days	Therapy success was achieved in 15 of 28 (53.5%) cases with CED and in 4 of 9 (44%) cases with corneal ulcers	Insulin drops were started after 36 days of conventional therapy

**Table 5:** Comparison of topical insulin vs. standard treatments.

Treatment	Mechanism of Action	Efficacy	Challenges
Artificial Tears	Hydration, mechanical protection	Symptomatic relief only	Does not promote healing
Growth Factors (NGF, EGF)	Stimulate epithelial and nerve regeneration	High (NGF shows nerve regeneration)	Expensive, limited access
Autologous Serum Eye Drops	Supply growth factors and anti-inflammatory cytokines	Moderate to high	Requires preparation from patient’s blood
Corneal Neurotization	Restores corneal sensation via nerve grafts	High (permanent effect)	Invasive, requires surgery
Topical Insulin	Stimulates epithelial proliferation, reduces inflammation	Promising (75–90% healing rates in studies)	Optimal dosage/duration not yet standardized

## Abbreviations

BCVA	best corrected visual acuity
CIPA	congenital insensitivity to pain
IGF	insulin growth factor
NGF	nerve growth factor
NK	neurotrophic keratitis
OCT	optical coherence tomography
PDGF	platelet-derived growth factor
RCTs	randomized clinical trials
TGF-β	transforming growth factor-beta
VEGF	vascular endothelial growth factor
